# Long-Term Coherent Integration Algorithm for High-Speed Target Detection

**DOI:** 10.3390/s24082603

**Published:** 2024-04-18

**Authors:** Yao He, Guanghui Zhao, Kai Xiong

**Affiliations:** 1 Guangzhou Institute of Technology, Xidian University, Guangzhou 510555, China; 2 School of Artificial Intelligence, Xidian University, Xi’an 710126, China; 3 Hangzhou Institute of Technology, Xidian University, Hangzhou 311231, China

**Keywords:** coherent detection, velocity estimation, range walk (RW) correction, high-speed target

## Abstract

Long-term coherent integration (CI) can effectively improve the radar detection capability for high-speed targets. However, the range walk (RW) effect caused by high-speed motion significantly degrades the detection performance. To improve detection performance, this study proposes an improved algorithm based on the modified Radon inverse Fourier transform (denoted as IMRIFT). The proposed algorithm uses parameter searching for velocity estimation, designs a compensation function based on the relationship between velocity and distance walk and Doppler ambiguity terms, and performs CI based on the compensated signal. IMRIFT can achieve RW correction, avoid the blind-speed sidelobe (BSSL) effect caused by velocity mismatch, and improve detection performance, while ensuring low computational complexity. In addition, considering the relationship between energy concentration regions and bandwidth in the 2D frequency domain, a fast method based on IMIRFT is proposed, which can balance computational cost and detection capacity. Finally, a series of comparative experiments are conducted to demonstrate the effectiveness of the proposed algorithm and the fast method.

## 1. Introduction

In recent years, the widespread application of high-speed targets in national defense and civilian fields has gained significant research interest in the radar field [1,2,3,4]. Considering the low radar cross-section (RCS) characteristics of these high-speed targets, increasing the integration time is an effective way to improve the signal-to-noise ratio (SNR) of an echo [5,6,7,8,9,10]. However, during the long-term coherent integration (CI) process, the range walk (RW) effect induced by high-speed targets renders the traditional CI method ineffective, significantly deteriorating radar detection performance [11,12]. Therefore, effective correction of the RW effect is necessary for improving radar detection performance.

To mitigate the RW effect induced by high-speed targets, many long-time coherent detection algorithms have been proposed, such as the Radon Fourier transform (RFT) [13,14], keystone transform (KT) [15,16], modified location rotation transform (MLRT) [17], scaled inverse Fourier transform (SCIFT) [18], and modified Radon inverse Fourier transform (MRIFT) [19]. Among these, the RFT algorithm achieves CI through a joint search in the range–velocity space. However, the presence of blind-speed sidelobe (BSSL) effects of RFTs lead to false alarms in detection. In addition, the KT algorithm is widely employed to mitigate the RW effect by scaling the slow-time dimension. However, the KT algorithm requires interpolation operations, which can cause significant computational burden and degradation of detection performance. The MLRT algorithm estimates the target velocity while correcting the RW effect by rotating the coordinates of the echo data. However, the rotation operation still imposes a significant computational burden. The SCIFT algorithm achieves CI by constructing autocorrelation functions, which greatly reduces the computational cost. However, the autocorrelation operation reduces the anti-noise ability and may cause the algorithm to fail under low-SNR conditions. MRIFT was proposed to improve the detection performance while reducing the computational cost. The MRIFT algorithm achieves CI by extracting lines in a two-dimensional frequency domain, performing phase compensation, and using IFT operations. However, owing to the influence of velocity mismatch, this algorithm also leads to detection performance loss.

Inspired by previous works, an improved modified Radon inverse Fourier transform (IMRIFT) is proposed in this study for high-speed targets. In the proposed algorithm, after obtaining the estimated velocity of the target through parameter estimation, a compensation function is constructed to correct the RW and Doppler ambiguity effects in the echo in the *t*-*f_m_* domain and the CI is achieved using FT and IFT.

Compared with [19], this study has been improved in the following aspects:

- The BSSL effect caused by velocity mismatch in [19] is analyzed and the IMRIFT can avoid the BSSL to improve detection performance;
- The results of line extraction operation in [19] are used to achieve velocity estimation and CI, while in IMRIFT, they are only used to obtain estimated velocity;
- A fast method for the IMRIFT is proposed, using only half the peak point compared to MRIFT to achieve velocity estimation.

Compared to other typical coherent detection algorithms, IMRIFT can avoid the BSSL effect and improve detection performance without increasing computational burden.

The remainder of this study is organized as follows. The signal model for high-speed targets is described in Section 2. Section 3 describes the proposed IMRIFT algorithm and the fast IMRIFT method. The numerical and simulation experiments are presented in Section 4. Section 5 summarizes the study.

## 2. Signal Model

Let us consider a pulse-Doppler radar system that transmits a linear frequency modulated (LFM) signal [13,20]

(1) $$s_{t}\left(t_{m},t\right)=\mathrm{rect}\left(\frac{t}{T_{P}}\right)\mathrm{rect}\left(\frac{t_{m}}{T_{CPI}}-0.5\right)\exp\left(j2\pi f_{0}t+j\pi \mu t^{2}\right)$$

where

(2) $$\mathrm{rect}\left(\frac{t}{T_{P}}\right)=\left\{\begin{array}{ll} 1 & \left|t\right|\leq 0.5T_{P}\\ 0 & \left|t\right|>0.5T_{P}. \end{array}\right.$$

Here, *μ* is the frequency modulation rate and *f*_0_ is the carrier frequency. *T_r_*, *T_p_*, and *t* correspond to the pulse repetition time, pulse duration, and fasting time, respectively. *t_m_* = *mT_r_* denotes the slow time (*m* = 0, 1, …, *M* − 1) where *M* is the pulse integration number. *T_CPI_* = *MT_r_* denotes one coherent processing interval (CPI) [20], indicating that the signal is zero when *m* ≥ *M* or *m* < 0.

We assume that there is a target with an initial radial range *R*_0_ and a constant radial velocity *v*_0_. The radial distance of the target at tm satisfies [13,14,15,16,17,18] the relation

(3) $$r\left(t_{m}\right)=R_{0}+v_{0}t_{m}.$$

The echo signal after the down-conversion and the pulse compression (PC) can be written as [7,20]

(4) $$s_{c}\left(t_{m},t\right)=AB\exp\left(-j4\pi \frac{R_{0}}{\lambda }\right)\mathrm{sinc}\left[B\left(t-2\frac{R_{0}+v_{0}t_{m}}{c}\right)\right]\exp\left(-j2\pi f_{d}t_{m}\right)\mathrm{rect}\left(\frac{t_{m}}{T_{CPI}}-0.5\right)$$

where *A* denotes the amplitude of the target after PC and sinc(*x*) = sin*πx*/(*πx*) denotes the sinc function. *B*, *λ*, and *c* denote the bandwidth of the transmitted signal, wavelength, and light speed, respectively. *f_d_* represents the Doppler frequency and is given as

(5) $$f_{d}=\frac{2v_{0}}{\lambda }=f_{d0}+G_{0}f_{r}$$

where *f_d_*_0_ is the ambiguous Doppler frequency, *G*_0_ is the Doppler ambiguity integer, and *f_r_* is the pulse repetition frequency (PRF).

From (5), it can be observed that the peak position of *s_c_* (*t_m_*, *t*) changes with slow time *t_m_*. When the offset exceeds the range resolution, i.e., Δ*r* = *c*/2*B*, RW would occur, which will interfere strongly with the coherent integration performance. Therefore, it is necessary to eliminate the RW effect before achieving long-time coherent integration.

## 3. Coherent Detection Based on Imrift

In this section, the MRIFT algorithm in [19] is introduced and its existing disadvantages are analyzed. In addition, the proposed algorithm (i.e., IMRIFT) is discussed in detail and its complexity is analyzed. Finally, a fast method for IMRIFT is provided.

### 3.1. Review of the MRIFT

By implementing the Fourier transform (FT) along the fast and slow time axes for the PC echoes in (4), we can obtain the 2D frequency domain echoes in the fast-slow frequency (*f*–*f_m_*) domain as follows:

(6) $$\begin{array}{ccc} S_{c}\left(f_{m},f\right) & = & AT_{CPI}\exp\left(-j4\pi R_{0}/\lambda \right)\exp\left(-j4\pi R_{0}f/c\right)\mathrm{rect}\left(f/B\right)\\ & & \cdot \exp\left[-j\pi T_{CPI}\left(f_{m}+f_{d}+2v_{0}f/c\right)\right]\mathrm{sinc}\left[T_{CPI}\left(f_{m}+f_{d}+2v_{0}f/c\right)\right] \end{array}$$

Here, *f* and *f_m_* represent fast and slow time frequency, respectively, and *f_d_* = 2*v*_0_*f*_0_/*c* is the ambiguous Doppler frequency of the target.

As shown in (6), the arrangement of the peaks of *S_c_* (*f_m_*, *f*) is presented as a straight line that satisfies the following equation:

(7) $$f_{m}=-f_{d}-2v_{0}f/c.$$

From (7), it can be observed that the variable of the line is *f* where velocity *v*_0_ is the only unknown parameter. Once velocity *v*_0_ is given, it is easy to calculate the position of the corresponding peak point (*f*, −2*v*_0_*f*_0_/*c* − 2*v*_0_*f*/*c*) using (7). By extracting these peak points in the *f*–*f_m_* domain, rearranging them along the fast time dimension, and performing the inverse Fourier transform (IFT) operation, the CI results can be obtained as follows:

(8) $$\begin{array}{ccc} s_{c}\left(v_{0},t\right) & = & IFT\left\{S_{c}\left(-2v_{0}f_{0}/c-2v_{0}f/c,f\right)\right\}\\ & = & ABT_{CPI}\exp\left(-j4\pi R_{0}/\lambda \right)\mathrm{sinc}\left[B\left(t-2\frac{R_{0}}{c}\right)\right]. \end{array}$$

In fact, owing to the influence of discrete sampling, the peak coordinates in (8) cannot be accurately extracted. Specifically, the sampling interval corresponding to the slow time frequency is *f_r_*/*M*. When utilizing (7) to calculate the slow time frequency at the peak position, the value of *f_m_* obtained is an integral multiple of *f_r_*/*M*, that is,

(9) $$f_{md}\left(v_{0},f\right)={\left[\left(-2v_{0}f/c-2v_{0}f_{0}/c\right)M/f_{r}\right]}_{r}f_{r}/M$$

where [·]_r_ is the rounding operator.

The error in the coordinate extraction process leads to amplitude and phase errors in the echo extraction operation, as follows:

(10) $$\begin{array}{ccc} S_{c}\left(v_{0},f\right) & = & S_{c}\left(f_{md}\left(v_{0},f\right),f\right)\\ & = & AT_{CPI}\exp\left(-j4\pi R_{0}/\lambda \right)\exp\left(-j4\pi R_{0}f/c\right)\\ & & \cdot \mathrm{rect}\left(f/B\right)\exp\left[-j\pi T_{CPI}\Delta f_{m}\left(v_{0},f\right)\right]\mathrm{sinc}\left[T_{CPI}\Delta f_{m}\left(v_{0},f\right)\right] \end{array}$$

(11) $$f_{mt}\left(v_{0},f\right)=-f_{d}-2v_{0}f/c$$

(12) $$\Delta f_{m}\left(v_{0},f\right)=f_{md}\left(v_{0},f\right)-f_{mt}\left(v_{0},f\right)$$

Here, *f_mt_* (*v*_0_, *f*) is the theoretical coordinate of *f_m_* for the peak point and Δ*f_m_* (*v*_0_, *f*) is the coordinate error of *f_m_*. To compensate for the phase and amplitude errors, a compensation function was designed in the MRIFT:

(13) $$S_{comp}\left(v_{0},f\right)=\exp\left[j\pi T_{CPI}\Delta f_{m}\left(v_{0},f\right)\right]/\sin c\left[T_{CPI}\Delta f_{m}\left(v_{0},f\right)\right].$$

After the compensation and IFT operations of (10), CI can be obtained as follows:

(14) $$\begin{array}{ccc} s_{c}\left(v_{0},t\right) & = & IFT\left\{S_{c}\left(v_{0},f\right)\cdot S_{comp}\left(v_{0},f\right)\right\}\\ & = & ABT_{CPI}\exp\left(-j4\pi R_{0}/\lambda \right)\mathrm{sinc}\left[B\left(t-2\frac{R_{0}}{c}\right)\right]. \end{array}$$

As shown in (14), the CI for the high-speed target eliminates the RW effect. The processes of line extraction, error compensation, and IFT are summarized in MRIFT. By utilizing velocity search operations, the velocity of the target and the CI can be achieved. The main steps of the process can be expressed as follows:

(15) $$s_{c}\left(v,t\right)=IFT\left\{S_{c}\left(f_{md}\left(v,f\right),f\right)\cdot S_{comp}\left(v,f\right)\right\}$$

(16) $$\left(v_{u,est},G_{est}\right)=\underset{v_{u},G}{\mathrm{argmax}}\left|s_{c}\left(v_{u}+Gv_{a},t\right)\right|$$

(17) $$v_{est}=v_{u,est}+G_{est}v_{a}$$

(18) $$s_{c}\left(v_{est},t\right)=IFT\left\{S_{c}\left(f_{md}\left(v_{est},f\right),f\right)\right.\left.\cdot S_{comp}\left(f_{md}\left(v_{est},f\right),f\right)\right\}$$

Here, $v_{u}\in \left[0,v_{a}\right)$ represents the unambiguous velocity and G∈Z is the ambiguity factor. *v_a_* = *f_r_λ*/2 is the basis of ambiguous velocity.

However, during the velocity search, MRIFT overlooks the mismatch between the actual target velocity and the measured velocity and because of this, the errors in (10) are not completely eliminated.

Assuming that *v_est_* is the measured velocity and *v*_0_ is the real velocity of the target. The compensation function in (13) should be modified as follows:

(19) $$S_{comp}\left(v_{est},f\right)=\frac{\exp\left[j\pi T_{CPI}\Delta f_{mr}\left(v_{est},f\right)\right]}{\mathrm{sinc}\left[T_{CPI}\Delta f_{mr}\left(v_{est},f\right)\right]}$$

(20) $$f_{mt}\left(v_{est},f\right)=-2v_{est}f_{0}/c-2v_{est}f/c$$

(21) $$\Delta f_{mr}\left(v_{est},f\right)=f_{md}\left(v_{est},f\right)-f_{mt}\left(v_{est},f\right).$$

Using the modified compensation function in (19) to compensate for the extracted echo in (10), the following result (22) is obtained.

(22) $$\begin{array}{ccc} S\left(v_{est},f\right) & = & S_{c}\left(v_{0},f\right)\cdot S_{comp}\left(v_{est},f\right)\\ & = & AT_{CPI}\exp\left(-j4\pi \frac{R_{0}}{\lambda }\right)\exp\left(-j4\pi \frac{R_{0}f}{c}\right)\mathrm{rect}\left(\frac{f}{B}\right)\\ & & \cdot \exp\left[-j\pi T_{CPI}\left(\Delta f_{m}\left(v_{est},f\right)-\Delta f_{mr}\left(v_{est},f\right)\right)\right]\\ & & \cdot \mathrm{sinc}\left[T_{CPI}\Delta f_{m}\left(v_{est},f\right)\right]/\mathrm{sinc}\left[T_{CPI}\Delta f_{mr}\left(v_{est},f\right)\right]\\ & = & AT_{CPI}\exp\left(-j4\pi \frac{R_{0}}{\lambda }\right)\exp\left(-j4\pi \frac{R_{0}f}{c}\right)\mathrm{rect}\left(\frac{f}{B}\right)\\ & & \cdot \exp\left[-j\pi T_{CPI}\Delta f_{mrt}\left(v_{est},f\right)\right]S_{\mathrm{sinc}}\left(f\right) \end{array}$$

(23) $$\Delta f_{m}\left(v_{est},f\right)=f_{md}\left(v_{est},f\right)-f_{mt}\left(v_{0},f\right)$$

(24) $$\begin{array}{ccc} \Delta f_{mrt}\left(v_{est},f\right) & = & f_{mt}\left(v_{est},f\right)-f_{mt}\left(v_{0},f\right)\\ & = & 2\left(v_{0}-v_{est}\right)\left(f+f_{0}\right)/c \end{array}$$

(25) $$S_{\mathrm{sinc}}\left(f\right)=\mathrm{sinc}\left[T_{CPI}\Delta f_{m}\left(v_{est},f\right)\right]/\mathrm{sinc}\left[T_{CPI}\Delta f_{mr}\left(v_{est},f\right)\right]$$

By performing the IFT operation on (22), the CI result of MRIFT is obtained as follows:

(26) $$\begin{array}{c} s_{c}\left(v_{est},t\right)=ABT_{CPI}\exp\left(-j4\pi R_{0}/\lambda \right)\mathrm{sinc}\left[B\left(t-2\frac{R_{0}}{c}-\frac{T_{CPI}\Delta v}{c}\right)\right]\\ \cdot \exp\left[-j2\pi T_{CPI}\Delta v/\lambda \right]⊗IFT\left\{S_{\mathrm{sinc}}\left(f\right)\right\} \end{array}$$

(27) $$\Delta v=v_{0}-v_{est}.$$

Compared to (14), the CI contains three error terms caused by the velocity mismatch in (26). Once *v*_0_ = *v_est_*, (26) yields the same CI result as (14).

The main impacts of these errors are discussed next. It should be clarified that the search velocity interval does not exceed the velocity resolution of the radar, while Δ*v* is typically small, not exceeding one search velocity interval. Therefore, *T_CPI_*Δ*v* < Δ*r*, indicating that the error term in the sinc function does not lead to the RW effect in CI.

The phase error term exp[−*j*2*πT_CPI_*Δ*v*/*λ*] causes a deviation in the phase of the CI result but does not cause RW and BSSL effects.

To better analyze the impact of amplitude error terms IFT{*S_sinc_*(*f*)}, it is necessary to simplify *S_sinc_*(*f*). Δ*f_mr_* (*v_est_*, *f*) and Δ*f_m_* (*v_est_*, *f*) can be calculated as follows:

(28) $$\begin{array}{ccc} \Delta f_{mr}\left(v_{est},f\right) & = & {\left[\left(-2v_{est}f/c-2v_{est}f_{0}/c\right)M/f_{r}\right]}_{r}f_{r}/M\\ & & +2v_{est}f/c+2v_{est}f_{0}/c\\ & = & \left\{\begin{array}{l} 2v_{est}f/c\;,\;b-\frac{cf_{r}}{4v_{est}M}<f\leq b+\frac{cf_{r}}{4v_{est}M}\\ \Delta f_{mr}\left(v_{est},f-nT_{est}\right)\;,\;n\in N \end{array}\right. \end{array}$$

(29) $$\begin{array}{ccc} \Delta f_{m}\left(v_{est},f\right) & = & {\left[\left(-2v_{est}f/c-2v_{est}f_{0}/c\right)M/f_{r}\right]}_{r}f_{r}/M\\ & & +2v_{0}f/c+2v_{0}f_{0}/c\\ & = & \left\{\begin{array}{l} 2v_{0}f/c+2\Delta vf_{0}/c\;,\;b-\frac{cf_{r}}{4v_{est}M}<f\leq b+\frac{cf_{r}}{4v_{est}M}\;\\ \Delta f_{m}\left(v_{0},f-nT_{est}\right)+nf_{r}\Delta v/\left(Mv_{est}\right),\;n\in N \end{array}\right. \end{array}$$

(30) $$T_{est}=\frac{cf_{r}N}{2Mv_{est}f_{s}}$$

(31) $$b={\left[-2v_{est}f_{0}/c\cdot M/f_{r}\right]}_{r}\cdot cf_{r}/\left(2v_{est}M\right)+f_{0}$$

Here, *T_est_* represents the periods of Δ*f_mr_* (*v_est_*, *f*) and Δ*f_m_* (*v*_0_, *f*).

Utilizing (28)–(31), the amplitude error term in (25) can be expressed as

(32) $$S_{\mathrm{sinc}}\left(f\right)=\left\{\begin{array}{l} S_{pe}\left(f\right)\;,\;b-\frac{cf_{r}}{4v_{est}M}<f\leq b+\frac{cf_{r}}{4v_{est}M}\;\mathrm{and}\;n=0\\ S_{\mathrm{sinc}}\left(f-nT_{est}\right)\;,\;n\in N \end{array}\right.$$

(33) $$\begin{array}{ccc} S_{pe}\left(f\right) & = & \frac{2v_{0}f/c+2\Delta vf_{0}/c+nf_{r}\Delta v/\left(Mv_{est}\right)}{2v_{est}f/c\;}\\ & & \cdot \frac{\sin\left[2T_{CPI}v_{est}f/c\right]}{\sin\left[T_{CPI}\left(2v_{0}f/c+2\Delta vf_{0}/c+nf_{r}\Delta v/\left(Mv_{est}\right)\right)\right]} \end{array}$$

Because 1/sin(*x*) does not satisfy the condition of absolute integrability in the entire real field, its IFT results are difficult to obtain [21]. Example 1 is provided to validate Equations (28)–(30).

**Figure 1:**
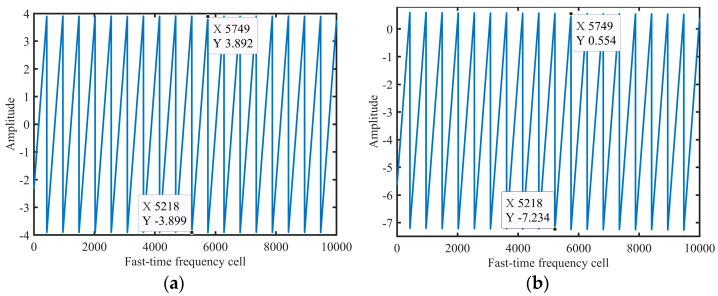

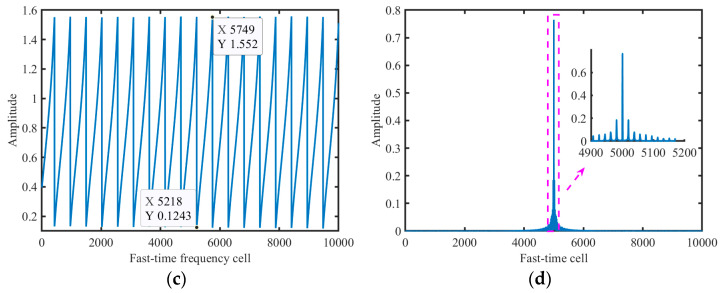
Simulation results of (30)–(33). Result of (**a**) Δ*f_mr_* (*v_est_*, *f*), (**b**) Δ*f_m_* (*v*_0_, *f*), and (**c**) *S_sinc_*(*f*). (**d**) IFT result of *S_sinc_*(*f*).

Example 1: Figure 1 shows the simulation results of (28)–(30). The radar parameters are listed in Table 1. To easily obtain the slope, we set *N* = 10000. The other parameters were set as *v*_0_ = 1100 m/s and *v_est_* = 1100.5 m/s.

Figure 1a,b show the results of Δ*f_mr_* (*v_est_*, *f*) and Δ*f_m_* (*v*_0_, *f*), respectively. Obviously, Δ*f_mr_* (*v_est_*, *f*) and Δ*f_m_* (*v*_0_, *f*) are periodic functions and the period 5749 − 5218 + 1 = 532 is approximately equal to *T_est_* = *cf_r_N*/(2*Mv_est_f_s_*) = 532.4284, calculated using (31). Additionally, the slope of Δ*f_mr_* (*v_est_*, *f*) in Figure 1a is (3.892 + 3.899)/(5749 − 5218) × *N*/*f_s_* = 7.3367 × 10^−6^, while the slope for Δ*f_m_* (*v_est_*, *f*) in Figure 1b is (0.554 + 7.234)/(5749 − 5218) × *N*/*f_s_* = 7.3333 × 10^−6^. The slopes calculated using (29) and (30) are 7.3367 × 10^−6^ and 7.3333 × 10^−6^, respectively, which proves the correctness of (29) and (31). Figure 1d shows the IFT result of *S_sinc_*(*f*).

Owing to the velocity mismatch, the presence of convolutional term IFT{*S_sinc_*(*f*)} in *s_c_* (*v_est_*, *t*) causes energy to spread in the target, leading to the BSSL effect. Decreasing the search velocity interval may result in the accurate estimation of the real velocity; however, it will inevitably prolong the search time and reduce operation efficiency. Therefore, a more suitable coherent detection method is required.

**Figure 2:**
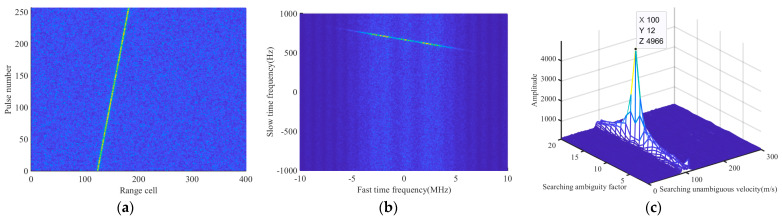

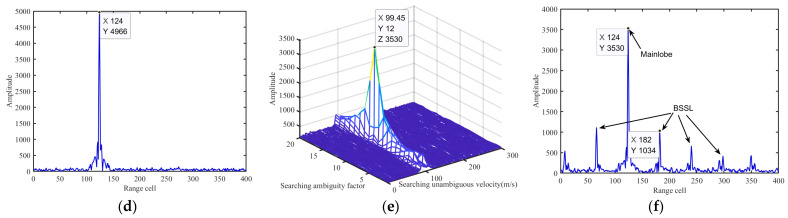
Results of MRIFT at different searching velocity intervals. (**a**) Echo after the PC result. (**b**) Signal echo in the 2D frequency domain. (**c**) Velocity search result of (**d**). (**d**) Velocity search interval is 1 m/s. (**e**) Velocity search result of (**f**). (**f**) Velocity search interval is 1.17 m/s.

Example 2: Figure 2 shows the results of the MRIFT algorithm at different search velocity intervals. The radar parameters are listed in Table 1. The simulation parameters of the target are as follows: *v*_0_ = 3400.1 m/s, *R*_0_ = 19.9225 km, and *A* = 1. The SNR of the echo received by the radar is −10 dB.

Figure 2a shows the results of the CI, indicating a severe RW effect caused by the high velocity of the target. Figure 2b shows the signal echo in the 2D frequency domain, which is clearly presented as a straight line. The CI results for a search velocity interval of 1 m/s are shown in Figure 2d. From the CI results, the estimated velocity was 3400 m/s, and the compensated error term was 0. The CI results when the searching velocity interval was 1.17 m/s are shown in Figure 2f. At this point, the estimated velocity was 3399.45 m/s. Compared to that observed in Figure 2c, there is a significant BSSL effect, as shown in Figure 2d.

### 3.2. IMRIFT for a Single Target

The BSSL caused by velocity mismatch in MRIFT can interfere with detection performance but has little impact on the accuracy of estimated velocity. Therefore, this study proposes the IMRIFT algorithm, which uses estimated velocity to correct RW and achieve CI, effectively avoiding the BSSL effect.

The FT is performed on *s_c_* (*t_m_*, *t*) along *t_m_*, which can be expressed as

(34) $$\begin{array}{ccc} S_{c}\left(t_{m},f\right) & = & A\exp\left(-j4\pi \frac{R_{0}}{\lambda }\right)\mathrm{rect}\left(\frac{f}{B}\right)\exp\left(-j4\pi \frac{R_{0}f}{c}\right)\\ & & \cdot \mathrm{rect}\left(\frac{t_{m}}{T_{CPI}}-0.5\right)\exp\left(-j2\pi f_{d}t_{m}\right)\exp\left(-j4\pi \frac{v_{0}t_{m}}{c}f\right). \end{array}$$

By comparing (4) and (34), it can be found that the presence of phase term exp(−*j*4*πv*_0_*t_m_f*/*c*) induces the RW effect and the presence of phase term exp(−*j*2*πf_d_t_m_*) induces the Doppler ambiguity effect in the slow-time frequency domain. Therefore, a compensation function was established as follows:

(35) $$S_{cor}(v,f)=\exp\left(j4\pi \frac{vt_{m}}{c}f\right)\cdot \exp\left(j2\pi \frac{2vf_{0}}{c}t_{m}\right).$$

Once the velocity of the target is obtained, (36) can be used to eliminate the RW and Doppler ambiguity effects in the signal. Assuming that the estimated velocity of the target obtained using the MRIFT algorithm is *v_est_*, compensating (34) yields.

(36) $$\begin{array}{ccc} S_{cc}\left(t_{m},f\right) & = & S_{c}\left(t_{m},f\right)\cdot S_{cor}(v_{est},f)\\ & = & A\exp\left(-j4\pi \frac{R_{0}}{\lambda }\right)\exp\left(-j4\pi \frac{R_{0}}{c}f\right)\mathrm{rect}\left(\frac{f}{B}\right)\mathrm{rect}\left(\frac{t_{m}}{T_{CPI}}-0.5\right)\\ & & \cdot \exp\left(-j2\pi t_{m}f\cdot \frac{2\left(v_{0}-v_{est}\right)}{c}\right)\exp\left(-j2\pi t_{m}f_{0}\cdot \frac{2\left(v_{0}-v_{est}\right)}{c}\right). \end{array}$$

After performing the IFT on *S_cc_* (*t_m_*, *f*) with respect to *f*, we obtain

(37) $$\begin{array}{ccc} S_{cc}\left(t_{m},t\right) & = & AB\exp\left(-j4\pi \frac{R_{0}}{\lambda }\right)\mathrm{sinc}\left[B\left(t-2\frac{R_{0}}{c}-2\frac{\left(v_{0}-v_{est}\right)t_{m}}{c}\right)\right]\\ & & \cdot \mathrm{rect}\left(\frac{t_{m}}{T_{CPI}}-0.5\right)\exp\left(-j2\pi t_{m}\frac{2\left(v_{0}-v_{est}\right)}{\lambda }\right) \end{array}$$

Because Δ*v* = *v*_0_ − *v_est_* does not exceed one searching velocity interval, *t_m_*Δ*v* < Δ*r*, which suggests that the RW effect caused by *t_m_*Δ*v* in the sinc function can be ignored. By performing the FT on (37) along tm, the CI result can be expressed as

(38) $$S_{CI}\left(f_{m},t\right)=ABT_{CPI}\exp\left(-j4\pi R_{0}/\lambda \right)\mathrm{sinc}\left[B\left(t-2\frac{R_{0}}{c}\right)\right]\mathrm{sinc}\left[T_{CPI}\left(f_{m}+\frac{2\left(v_{0}-v_{est}\right)}{\lambda }\right)\right]$$

Although there is still an error term caused by the velocity mismatch in the CI result of (38), this error term causes only the peak point coordinates of the target to shift by the fm dimension. If Δ*v* < *v_a_*, it will not cause the Doppler ambiguity effect. In addition, the estimated velocity of the target can be subjected to secondary estimation. Assuming that the slow time frequency of the peak steaming point is *f_m_*_0_, the velocity of the secondary estimation is

(39) $$v_{se}=v_{est}+f_{m0}\cdot \lambda /2$$

**Figure 3:**
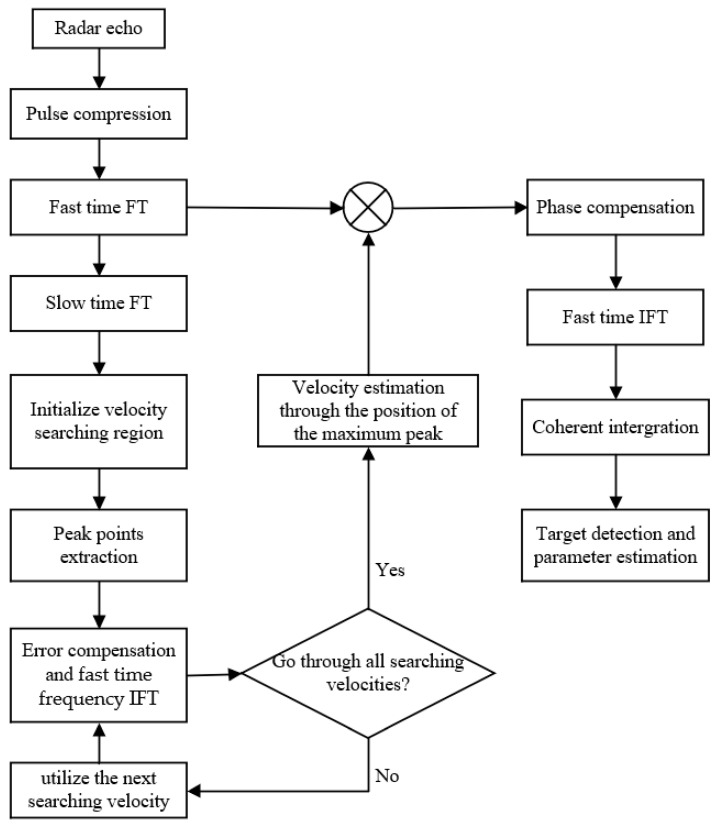
Flowchart of the IMRIFT.

The IMRIFT algorithm is illustrated in Figure 3.

Although the CI results of the IMRFT and MRIFT algorithms both include error terms caused by velocity mismatch, the effects caused by these error terms are completely different. In the CI result of MRIFT, e.g., (26), the error term exists in the form of a convolution, which can lead to the spreading of the peak energy of the target and the BSSL effect. However, in the CI result of IMRIFT, e.g., (38), the error term exists in the form of multiplication, which changes only the peak position of the target without causing any spreading of the peak energy. Therefore, compared to MRIFT, IMRIFT can avoid the BSSL effect.

**Figure 4:**
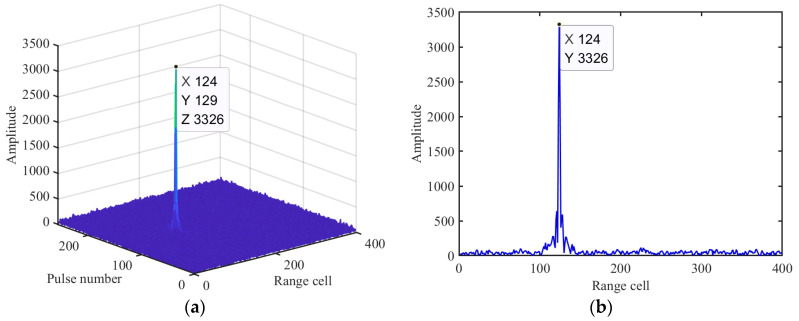
Results of IMRIFT. (**a**) CI result. (**b**) Slice with a pulse number of 129.

To verify the effectiveness of IMRIFT, a simulated example is provided. The parameters of the radar and target are the same as those described in Example 2 and the search velocity interval is 1.17 m/s. Figure 4a shows the CI results for the proposed IMRIFT. Figure 4b shows the CI results of the range cell with 129 pulses. Figure 4b shows that after the IMRIFT, the RW and Doppler ambiguity effects were accurately removed. In addition, compared with those in Figure 2d, the side lobes in the CI result obtained by the IMRIFT were significantly suppressed.

### 3.3. IMRIFT for Multiple Targets

We assume that *K* targets move at a constant radial velocity in the scene. The PC signal can be expressed as

(40) $$s_{mc}\left(t_{m},t\right)=\sum_{k=1}^{K}A_{k}B\exp\left(-j4\pi \frac{R_{k}}{\lambda }\right)\exp\left(-j2\pi f_{dk}t_{m}\right)\mathrm{sinc}\left[B\left(t-2\frac{R_{k}+v_{k}t_{m}}{c}\right)\right]\mathrm{rect}\left(\frac{t_{m}}{T_{CPI}}-0.5\right)$$

where *A_k_*, *R_k_*, *v_k_*, and *f_dk_* represent the amplitude, initial radial range, radial velocity, and Doppler frequency of the *k*th target, respectively.

After conducting the FT along the fast time, *s_mc_* (*t_m_*, *t*) can be expressed as

(41) $$\begin{array}{ccc} S_{mc}\left(t_{m},f\right) & = & \sum_{k=1}^{K}A_{k}\exp\left(-j4\pi \frac{R_{k}}{\lambda }\right)\mathrm{rect}\left(\frac{t_{m}}{T_{CPI}}-0.5\right)\\ & & \cdot \mathrm{rect}\left(\frac{f}{B}\right)\exp\left(-j4\pi \frac{R_{k}f}{c}\right)\exp\left(-j2\pi f_{dk}t_{m}\right)\exp\left(-j4\pi \frac{v_{k}t_{m}}{c}f\right) \end{array}$$

It is assumed that the radial velocity of the ith target is *v_i_* and the estimated velocity is *v_i_est_*, where *v_i_* − *v_i_est_* does not exceed one searching velocity interval, respectively.

By correcting the RW and Doppler ambiguities caused by the ith target according to (35), we obtain

(42) $$\begin{array}{ccc} S_{mf}\left(t_{m},f\right) & = & A_{i}\exp\left(-j4\pi \frac{R_{i}}{\lambda }\right)\mathrm{rect}\left(\frac{f}{B}\right)\mathrm{rect}\left(\frac{t_{m}}{T_{CPI}}-0.5\right)\\ & & \cdot \exp\left(-j4\pi \frac{R_{i}f}{c}\right)\cdot \exp\left(-j2\pi t_{m}f_{di}\cdot \frac{2\left(v_{i}-v_{i\_est}\right)}{c}\right)+S_{other}\left(t_{m},f\right) \end{array}$$

where

(43) $$\begin{array}{ccc} S_{other}\left(t_{m},f\right) & = & \sum_{k=1,k\neq i}^{K}A_{k}\exp\left(-j4\pi \frac{R_{k}}{\lambda }\right)\mathrm{rect}\left(\frac{f}{B}\right)\mathrm{rect}\left(\frac{t_{m}}{T_{CPI}}-0.5\right)\\ & & \cdot \exp\left(-j4\pi \frac{R_{k}f}{c}\right)\exp\left[-j4\pi \left(v_{k}-v_{i\_est}\right)t_{m}f/c\right]\exp\left[-j2\pi \left(f_{dk}-f_{di}\right)t_{m}\right] \end{array}$$

By performing fast-time and slow-time FT, the CI result of the *i*th target can be represented as follows:

(44) $$\begin{array}{ccc} S_{mc}\left(f_{m},t\right) & = & A_{i}BT_{CPI}\exp\left(-j\pi T_{CPI}f_{m}\right)\mathrm{sinc}\left[B\left(t-\frac{2R_{i}}{c}\right)\right]\\ & & \cdot \mathrm{sinc}\left[T_{CPI}\left(f_{m}+\frac{2\left(v_{i}-v_{i\_est}\right)}{\lambda }\right)\right]+S_{other}\left(f_{m},t\right)\\ S_{other}\left(f_{m},t\right) & = & \underset{t_{m}}{FFT}\left\{\underset{f}{IFFT}\left\{S_{other}\left(t_{m},f\right)\right\}\right\} \end{array}$$

### 3.4. Computational Complexity Analysis

In this subsection, we analyze the computational complexity of the main steps in the proposed algorithm. The KT [16], RFT [13], MLRT [17], SCIFT [18], and MRIFT [19] algorithms were compared. The number of echo pulses, number of range cells, search ambiguity factor, and search ambiguity velocity are *M*, *N*, *N_G_*, and *N_S_*, respectively.

For IMRIFT, the computational complexity of a certain velocity estimation is *O* [0.5*MN*(log_2_*M* + log_2_*N*) + *N_s_*(0.5*N*log_2_*N* + *N*))]. For velocity compensation and CI, *O* [0.5*MN*(log_2_*M* + log_2_*N*)] is needed. Therefore, the complexity of IMRIFT can be represented as O[*MN*(log_2_*M* + log_2_*N*) + *N_s_*(0.5*N*log_2_*N* + *N*))].

The main steps contain searching for the fold factor, sinc interpolation, and FT operation. Hence, the computational complexity was *O*(*N_G_NMM*). Because of the 2D search in terms of range and velocity, the computational cost of RFT is *O*(*N_s_NM*). For MLRT, angle searching is required; therefore, its computational complexity is *O*(*N_s_NM*). In addition, SCIFT has a computational cost of *O*(*NMM*) for the symmetric autocorrelation operation. In terms of MRIFT, the computational complexity is *O*[(*N_s_* + 1)(0.5*N*log_2_*N* + *N*) + 0.5*MN*(log_2_*M* + log_2_*N*)] because of velocity research and error compensation.

The computational complexity and actual processing time of the aforementioned method are listed in Table 2. The parameters of the radar and target are the same as those in Example 2. The time overhead of IMRIFT is less than those of KT, RFT, MLRT, and SCIFT, indicating that IMRIFT is more efficient than these methods. However, the time complexity of IMRIFT was slightly higher than that of MRIFT.

### 3.5. A Fast Implementation Method for IMRIFT

In the implementation process of IMRIFT, multiple line extractions and IFT operations are required to obtain the estimated velocity of the target. To improve the efficiency of IMRIFT, we propose a fast implementation method to optimize the velocity estimation process.

The Nyquist–Shannon sampling theorem states that to accurately sample and reconstruct a signal, the sampling frequency must be equal to or greater than twice the signal’s bandwidth [22,23]. In this study, the sampling frequency is set as *f_s_* = 2*B*, which means the range of fast time-frequency f is from −*B* to *B*.

For (7), the presence of the rectangular window function rect(*f*/*B*) causes the energy of the echo signal in the 2D frequency domain to be concentrated within the range of −0.5*B* to 0.5*B*. Therefore, performing line extraction operations within the range of −0.5*B* to 0.5*B* can accurately obtain the energy of the echo signal, without the need for line extraction operations throughout the fast-time dimension. At this point, the main idea of the fast method of IMRIFT is to extract peak points within the range of −0.5*B* to 0.5*B* during the velocity estimation process to perform IFT operations and count the peaks in the IFT results for velocity estimation.

The process of velocity estimation in the fast method based on IMRIFT is as follows:

(45) $$s_{q}\left(v,t\right)=\int_{-0.5B}^{0.5B}S_{c}\left(f_{md}\left(v,f\right),f\right)S_{comp}\left(v,f\right)\exp(j2\pi ft)df$$

(46) $$\left(v_{u,est},G_{est}\right)=\underset{v_{u},G}{\mathrm{argmax}}\left|s_{q}\left(v_{u}+Gv_{a},t\right)\right|$$

(47) $$v_{est}=v_{u,est}+G_{est}v_{a}$$

where the resolution of *t* is 2/*f_s_*. After obtaining the estimated velocity *v_est_*, CI can be achieved using (36)–(39).

For the fast method based on IMRIFT, during the velocity searching process, only 0.5*N* points in the fast time-frequency dimension are required, resulting in a time cost of O[*N_s_*(0.5log_2_0.5*N* + 0.5*N*)] for velocity searching. Therefore, the time cost of the fast method is denoted as O[*MN*(log_2_*M* + log_2_*N*) + *N_s_*(0.5*N*log_2_0.5*N* + 0.5*N*)].

**Figure 5:**
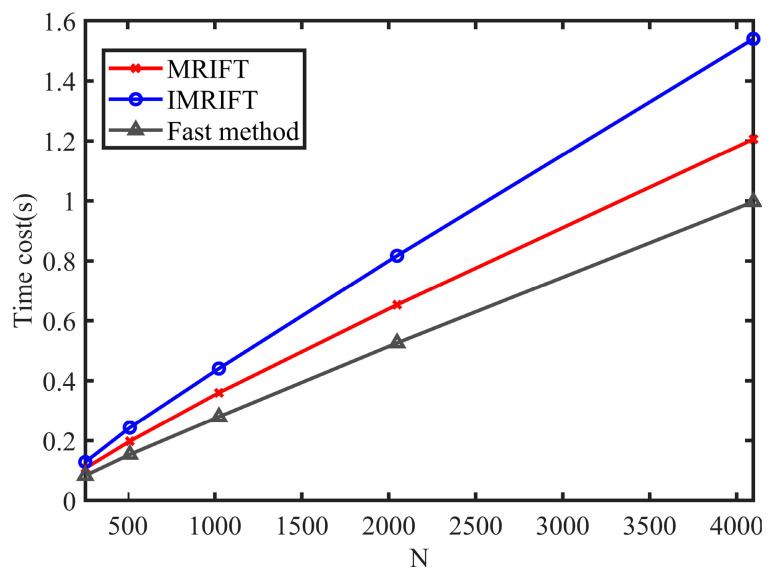
Computational costs of the fast method, IMRIFT, and MRIFT.

A comparison of the time costs of the fast method, MIRFT, and IMRIFT is shown in Figure 5. It can be observed that as the number of range cells *N* increases, the fast method has a lower time cost compared to MRIFT and IMIRFT.

**Figure 6:**
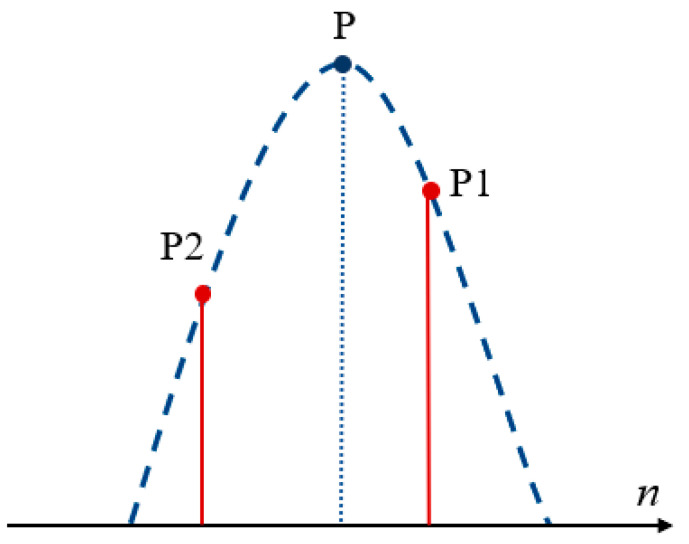
The relationship between the peak point, P1, and P2.

However, note that there may be peak information loss when using these 0.5*N* points to achieve the IFT. The peak position of the IFT results corresponds to the initial range cell of the target. The IFT result obtained by the fast method only consists of 0.5*N* points, which is lesser than the number for IMRIFT and equivalent to losing half of the range cell. Assuming that the search velocity is known, the IFT result of the fast method in velocity estimation is *s_cn_*(*n_new_*), where *n* = 0, 2, 4, …, *N* − 1. When the initial range cell of the target is odd, the fast method obtains the maximum value of point P1 using *s_cn_*(*n_new_*), instead of the peak point. As shown in Figure 6, the peak point was located between P1 and the second maximum value point P2 and the amplitude was higher than that of P1. Therefore, in this case, the maximum value of *s_cn_*(*n_new_*) obtained at different search velocities will have varying degrees of amplitude loss, which could lead to errors in the velocity estimation result and thus affect the detection performance of the fast method. The detection performance of the fast method was equivalent to that of IMRIFT when the initial distance unit of the target was even.

## 4. Experimental Results

Several numerical experiments were conducted to verify the effectiveness of the proposed IMRIFT algorithm. The parameters of the radar are listed in Table 1. In addition, comparisons with other common coherent integration algorithms such as KT, RFT, MLRT, SCIFT, and MRIFT are provided.

### 4.1. Coherent Integration for a Single Target

**Figure 7:**
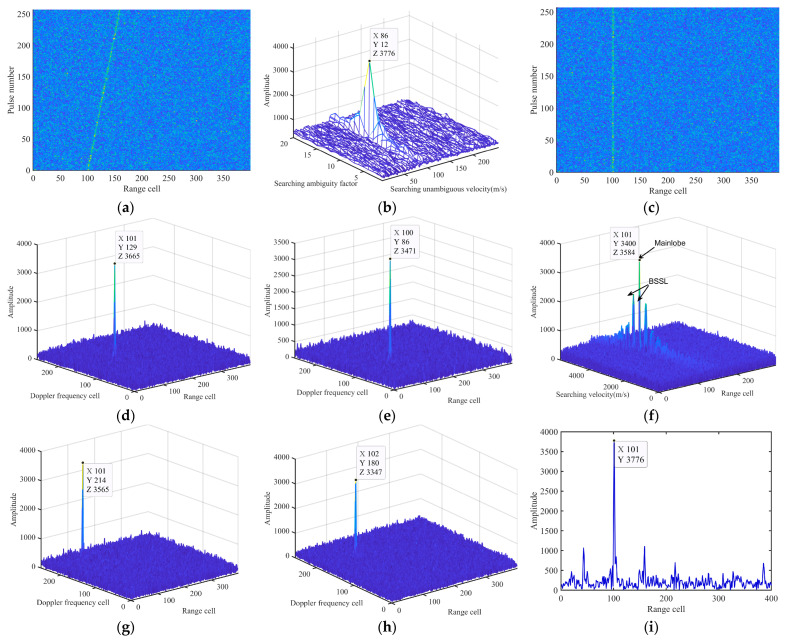
CI results for a single target via several other methods. (**a**) Echo after PC. (**b**) Velocity search result of IMRIFT. (**c**) Distance walk correction result of IMRIFT. (**d**) CI result for IMRIFT. (**e**) CI result for KT. (**f**) CI result for RFT. (**g**) CI result for MLRT. (**h**) CI result for SCIFT. (**i**) CI result for MRIFT.

To evaluate the performance of the IMRIFT in the case of a single target, the results of CI were obtained as shown in Figure 7. The motion parameters of the target were as follows: *A* = 1, *v*_0_ = 3400.1 m/s, and *R*_0_ = 19.75 km. The received signal is contaminated by zero-mean Gaussian white noise with an SNR of −25 dB.

Figure 7a shows the PC results in which severe RW effects can be observed. Figure 7b presents the velocity estimation results obtained using the velocity search method. The velocity was estimated as 3399.61 m/s based on (18). Figure 7c shows the result of the RW correction based on the measured velocity and Figure 7d displays the CI results of IMRIFT, where the energy accumulation of the target appears as a distinct peak (3665).

We compared the CI results of five methods: KT, RFT, MLRT, SCIFT, and MRIFT. Figure 7e–i shows the CI results of the five methods. The integration amplitudes of KT (3471), RFT (3584), and MLRT (3565) were lower than those of IMRIFT (3665). Furthermore, a clear effect of BSSL was observed in the RFT results. Figure 7i shows the results of MRIFT. The integration amplitude of the MRIFT (3776) is slightly higher than that of the IMRIFT.

### 4.2. Coherent Integration for Multiple Targets

**Figure 8:**
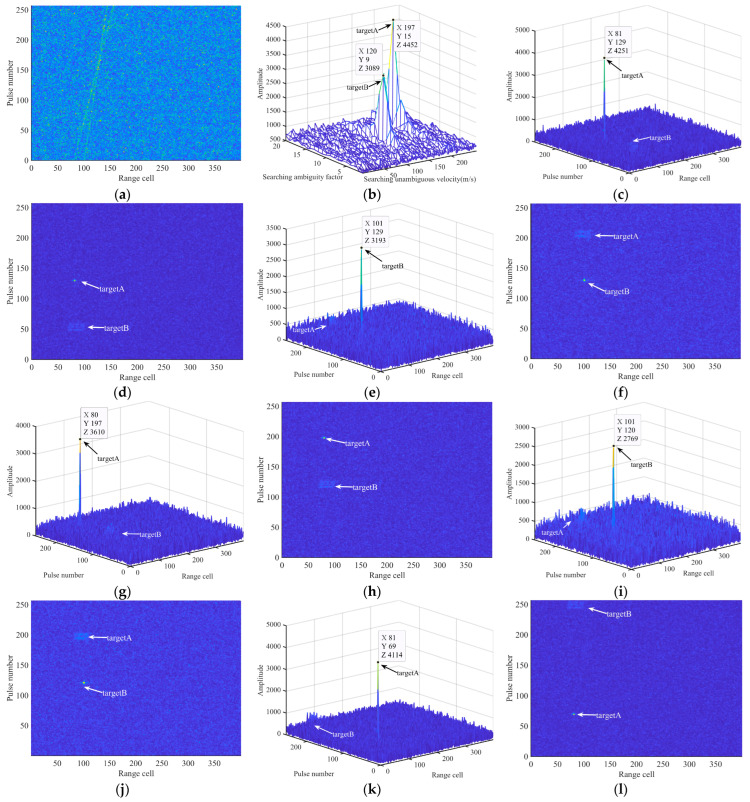

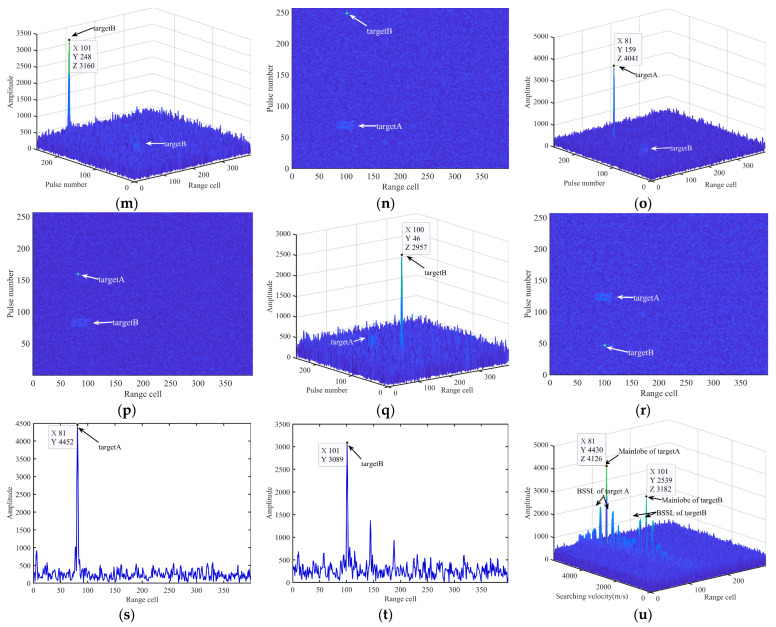
CI for multitarget via several other methods. (**a**) Echo after PC. (**b**) Velocity searching result of IMRIFT. (**c**) CI result of IMRIFT for targetA. (**d**) CI result of IMRIFT for targetA. (**e**) CI result of IMRIFT for targetB. (**f**) CI result of IMRIFT for targetB. (**g**) CI result of KT for targetA. (**h**) CI result of KT for targetA. (**i**) CI result of KT for targetB. (**j**) CI result of KT for targetB. (**k**) CI result of MLRT for targetA. (**l**) CI result of MLRT for targetA. (**m**) CI result of MLRT for targetB. (**n**) CI result of MLRT for targetB. (**o**) CI result of SCIFT for targetA. (**p**) CI result of SCIFT for targetA. (**q**) CI result of SCIFT for targetB. (**r**) CI result of SCIFT for targetB. (**s**) CI result of MRIFT for targetA. (**t**) CI result of MRIFT for targetB. (**u**) CI result of RFT.

The results of multitarget CI using the proposed algorithm and other methods are shown in Figure 8. Table 3 lists the motion parameters of Targets A and B. The SNR of the received echo signal is −20 dB. Figure 8a shows the results of PC and Figure 8b presents the results of the velocity search, from which the velocities can be calculated as 4429.69 m/s (target A) and 2539.45 m/s (Target B). The CI results of IMRIFT, KT, MLRT, and SCIFT are shown in Figure 8c–u. It can be seen that all four algorithms achieved CI for both targets. Figure 8s,t shows the results of MRIFT. It can be seen that the energy of both Targets A and B accumulates well. However, there is a strong sidelobe in the CI result of Target B, which may affect the detection performance of multiple targets. Figure 8u shows the CI results of RFT, in which there is an obvious BSSL effect. The “CLEAN” [24,25,26] method is usually used to suppress this effect.

### 4.3. Detection and Parameters Estimation Performance

In this experiment, Monte Carlo simulations were used to analyze the detection, range estimation, and velocity estimation performances of KT, RFT, MLRT, SCIFT, MRIFT, IMRIFT, and fast IMRIFT methods. The false alarm probability was set to P*_fa_* = 10^−3^ and the SNR was varied from −50 dB to −10 dB. Five hundred experiments were conducted for each scenario. The motion parameters of the target were set as *A* = 1, *v*_0_ = 3400.1 m/s, and *R*_0_ = 19.9225 km. The range cell number of *R*_0_ is 123.

**Figure 9:**
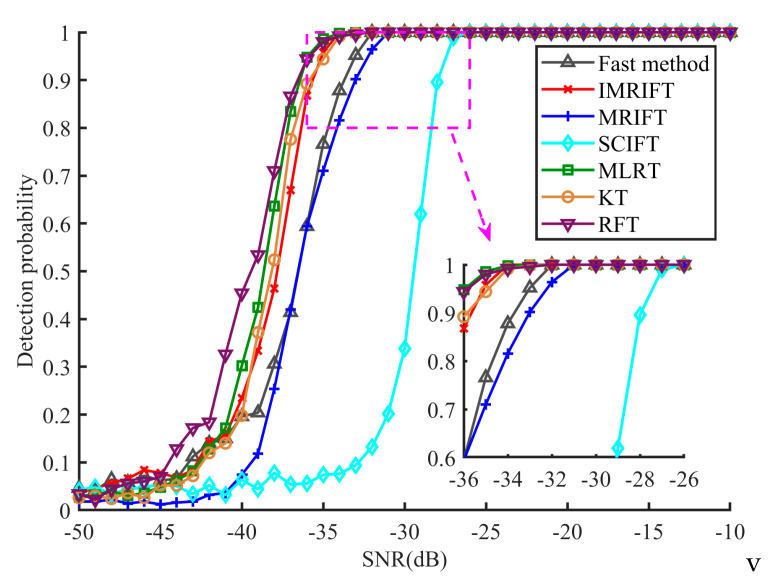
Detection probability of KT, RFT, MLRT, SCIFT, MRIFT, IMRIFT, and the fast method.

Figure 9 shows the detection probabilities of the seven methods at different SNR levels. The results indicate that IMRIFT has better detection performance than MRIFT and SCIFT; this performance is comparable to that of KT. Additionally, the proposed method suffers some performance losses in low-SNR scenarios compared with the case of MLRT. In the case of fast IMRIFT, there is a 2 dB loss in detection performance compared to that of IMRFT, while there is still a 1 dB advantage compared to that of MIRFT.

**Figure 10:**
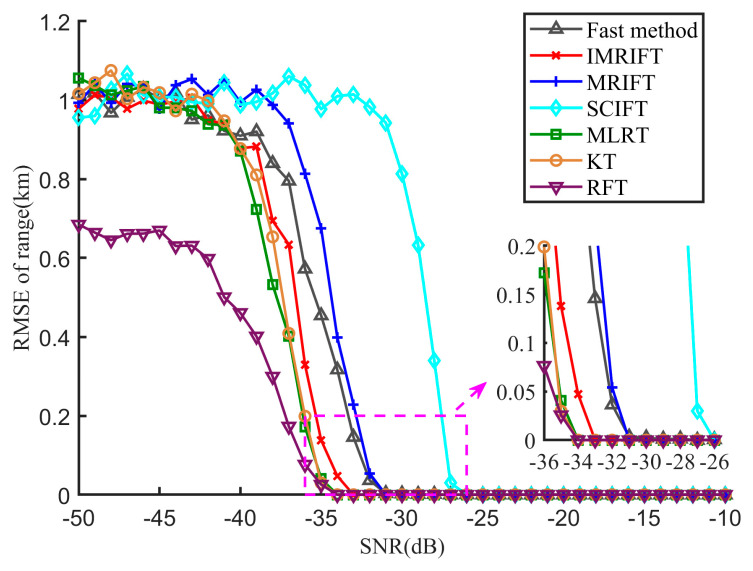
Range estimation performance of KT, RFT, MLRT, SCIFT, MRIFT, MRIFT, and the fast method.

The RMSE results for this range are shown in Figure 10. When the SNR ≥ −33 dB, the estimation error of IMRIFT is zero. Meanwhile, when the SNR ≥ −31 dB, the estimation error of MRIFT zero. In other words, IMRIFT obtains more accurate range estimates than MRIFT. However, compared with the cases of KT, RFT, and MLRT, there was still a 2dB performance loss. In addition, the performance of the fast method is comparable to that of MRIFT in terms of range estimation. Fast IMRIFT has a 2 dB loss in range performance compared to IMRFT, while its performance is comparable to that of MRIFT in terms of the range estimation performance.

**Figure 11:**
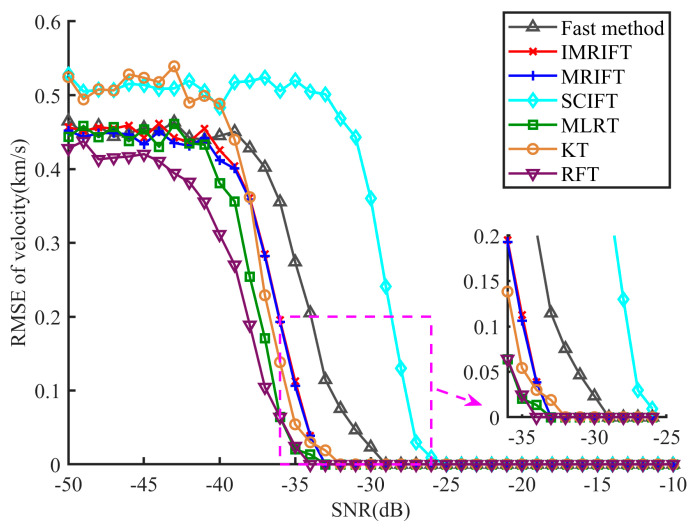
Velocity estimation performance of KT, RFT, MLRT, SCIFT, IMRIFT, and the fast method.

Figure 11 shows the RMSE results for the velocity. Because IMRIFT and MRIFT use the same method for velocity estimation, their velocity mean square errors are comparable. From Figure 11, it can be seen that the velocity estimation performance of IMRIFT is similar to that of KT and MLRT and better than that of SCIFT. However, compared with RFT, IMRIFT still exhibits a 2 dB performance loss. In addition, compared to IMRIFT, the fast method has a 4 dB loss in velocity estimation but the velocity estimation performance is still better than that of SCIFT.

## 5. Conclusions

In this study, an effective coherent detection algorithm, IMRIFT, was proposed for high-speed moving targets. In this method, the RW is corrected using the target estimated velocity obtained from the velocity search in the 2-D frequency domain; subsequently, a slow-time FT is performed to realize the CI. Compared with MRIFT, the proposed IMRIFT effectively avoids the BSSL effect, while maintaining better detection performance with a slight increase in computational cost. In addition, to reduce the time complexity of IMRIFT, this study proposes a fast method based on IMRIFT. This method can effectively reduce computational cost but its performance decreases when the initial range units of the target are odd. The experimental results demonstrate the effectiveness of IMRIFT and the fast method.

## Figures and Tables

**Table 1:** Parameters of radar.

Carrier frequency *f*_0_	1 GHz
Bandwidth *B*	10 MHz
Sample frequency *f_s_*	20 MHz
Pulse repetition frequency *f_r_*	2 kHz
Pulse duration *T_p_*	10^−6^ s
Number of pulses *M*	256
Number of range cells *N*	400

**Table 2:** Computational complexity.

Algorithm	Complexity	Time Cost (s)
KT	$O\left(N_{G}NMM\right)$	9.4956
RFT	$O\left(N_{S}NM\right)$	12.0155
MLRT	$O\left(N_{S}NM\right)$	11.9839
SCIFT	$O\left(NNM\right)$	0.3248
MRIFT	$O\left[\begin{array}{l} \left(N_{s}+1\right)\left(0.5N\log_{2}N+N\right)\\ +0.5MN\left(\log_{2}M+\log_{2}N\right) \end{array}\right]$	0.0655
IMRIFT	$O\left[\begin{array}{l} N_{s}\left(0.5N\log_{2}N+N\right)\\ +MN\left(\log_{2}M+\log_{2}N\right) \end{array}\right]$	0.0821

Configuration of the computer: CPU: Intel Core i9-10900K 3.7 GHz; RAM: 32 GHz; Operating System: Windows 10; Software: MATLAB R2021b.

**Table 3:** Motion parameters of two targets.

Target	Initial Range (km)	Velocity (m/s)
A	19.60	4430
B	19.75	2540

## Data Availability

Data are contained within the article.
